# The Graduated Embryo Score of Embryos from Infertile Women with and without Peritoneal Endometriosis

**DOI:** 10.1055/s-0040-1721855

**Published:** 2021-01-29

**Authors:** Juliana Caran, Vanessa Krebs Genro, Carlos Augusto Bastos de Souza, João Sabino Cunha-Filho

**Affiliations:** 1 Hospital de Clínicas de Porto Alegre, Universidade Federal do Rio Grande do Sul, Porto Alegre, RS, Brazil; 2 Department of Obstetrics and Gynecology, Centro de Reprodução Humana Insemine, Porto Alegre, RS, Brazil

**Keywords:** endometriosis, in vitro fertilization, graduated embryo score, infertility, endometriose, fertilização in vitro, escore do embrião, infertilidade

## Abstract

**Objective** To determine embryo quality (mean graduated embryo score [GES]) in infertile patients with endometriosis undergoing in vitro fertilization with embryo transfer (IVF-ET) compared with infertile patients without endometriosis.

**Methods** A case-control study was performed comparing 706 embryos (162 patients) divided into 2 groups: 472 embryos derived from patients without endometriosis ( *n* = 109, infertile patients with tubal infertility) and 234 embryos from patients in the study group ( *n* = 53, infertile patients with peritoneal endometriosis). All patients were subjected to IVF using an oestradiol-antagonist-recombinant follicle-stimulating hormone (FSH) protocol for ovarian stimulation. The mean GES was performed to evaluate all embryos at 3 points in time: 16 to 18 hours, 25 to 27 hours, and 64 to 67 hours. Embryo evaluation was performed according to the following parameters: fragmentation, nucleolar alignment, polar body apposition, blastomere number/morphology, and symmetry. The primary outcome measure was the mean GES score. We also compared fertilization, implantation, and pregnancy rates.

**Results** Although the number of embryos transferred was greater in patients with endometriosis than in the control group (2.38 ± 0.66 versus 2.15 ± 0.54; *p* = 0.001), the mean GES was similar in both groups (71 ± 19.8 versus 71.9 ± 23.5; *p* = 0.881). Likewise, the fertilization rate was similar in all groups, being 61% in patients with endometriosis and 59% in the control group ( *p* = 0.511). No significant differences were observed in the implantation (21% versus 22%; [ *p* = 0.989]) and pregnancy rates (26.4% versus 28.4%; *p* = 0.989).

**Conclusion** Embryo quality measured by the mean GES was not influenced by peritoneal endometriosis. Likewise, the evaluated reproductive outcomes were similar between infertile patients with and without endometriosis.

## Resumo

**Objetivo** Determinar a qualidade do embrião (média de escore embrionário graduado [EEG]) em pacientes inférteis com endometriose submetidas à fertilização in vitro com transferência de embrião (FIV-TE) em comparação com pacientes inférteis sem endometriose.

**Métodos** Realizamos um estudo de caso-controle comparando 706 embriões (162 pacientes) divididos em dois grupos: 472 embriões derivados de pacientes sem endometriose ( *n* = 109, pacientes inférteis com infertilidade tubária) e 234 embriões de pacientes do grupo de estudo ( *n* = 53, inférteis pacientes com endometriose peritoneal). Todos os pacientes foram submetidos à fertilização in vitro usando um protocolo *follicle-stimulating hormone* (FSH) recombinante de estradiol-antagonista para estimulação ovariana. A média do EEG foi realizada para avaliar todos os embriões em três momentos: de 16 a 18 horas, 25 a 27 horas e 64 a 67 horas. A avaliação embrionária foi realizada de acordo com os seguintes parâmetros: fragmentação, alinhamento nucleolar, aposição do corpo polar, número de blastômeros/morfologia e simetria. A medida de desfecho primário foi o escore médios embrionário (EEG). Também avaliamos como desfechos secundários as taxas de fertilização, implantação e gravidez.

**Resultados** Embora o número de embriões transferidos tenha sido maior em pacientes com endometriose do que no grupo controle (2,38 ± 0,66 versus 2,15 ± 0,54; *p* = 0,001), o EEG médio foi semelhante nos dois grupos (71 ± 19,8 versus 71,9 ± 23,5; *p* = 0,881). Da mesma forma, a taxa de fertilização foi semelhante em todos os grupos, sendo 61% nos pacientes com endometriose e 59% no grupo controle ( *p* = 0,511). Não foram observadas diferenças significativas nas taxas de implantação (21% versus 22%; [ *p* = 0,989]) e nas taxas de gravidez (26,4% versus 28,4%; *p* = 0,989).

**Conclusão** A qualidade embrionária medida pelo EEG médio não foi influenciada pela endometriose peritoneal. Da mesma forma, os resultados reprodutivos avaliados foram semelhantes entre pacientes inférteis com e sem endometriose.

## Introduction

In vitro fertilization with embryo transfer (IVF-ET) has been prescribed in many cases of endometriosis-associated infertility. This process has enabled us to study how this disease affects a patient's fertility and IVF outcomes. 1 2 3

Although previous studies on IVF outcomes have produced controversial results, it has been shown that patients with endometriosis have altered hormonal status, luteal phase deficiency, and folliculogenesis, as well as oocyte dysfunction, which may affect their embryo quality. 4 5 6 7 8 Recently, several new studies have failed to identify a clear link between endometriosis and decreased IVF results. 3 9 10

Embryo quality evaluation for the selection and transfer into the uterus is a critical step in IVF treatment to maximize the probability of pregnancy, especially when we choose to transfer only one embryo. 11 12 Previous retrospective studies have evaluated embryo quality in patients with endometriosis and observed a decrease in their morphological parameters, as well as a higher percentage of abnormal embryos in these patients. 13 14 Furthermore, several recent studies have shown a decreased oocyte quality and number in endometriotic patients. Nevertheless, when analyzing embryo development, the effect of endometriosis has not been thoroughly elucidated to date. 15 16

The graduated embryo score (GES) is a system of classification that relies on a combination of pronuclear morphology, early cleavage and day 3 morphology used to identify which embryos have a high potential for blastocyst conversion, as well as a higher probability of implantation and pregnancy. The present system is based on three evaluations for embryo assessment after insemination and awards a total possible score of 100 points. 12

The primary objective of this study was to verify if the embryo quality based on the mean GES scoring is different between infertile patients with peritoneal endometriosis and patients in the control group (tubal infertility). In addition, as secondary objectives, we compared several reproductive outcomes, such as implantation, fertilization, and pregnancy rates, between the groups.

## Methods

### Design

We performed a prospective case-control study.

### Setting

Infertile patients undergoing in vitro fertilization treatment at the Insemine Center for Human Reproduction, in Brazil, were recruited from 2010 to 2015. The patients were followed-up during the controlled ovarian stimulation until 12 days after the embryo transfer (first pregnancy test), and data collection was performed in the initial assessment (first ultrasound scan) and the laboratory results (embryo score as described below).

### Patients/Methods

A total of 162 women who underwent IVF-ET for the treatment of infertility in our unit were included. The patients were divided into two groups according to the presence (study group) or absence (control group, tubal occlusion) of endometriosis, after signing the free informed consent form. The eligibility criteria were: infertile women with endometriosis or tubal factor submitted to IVF for the first time, ages between 20 and 40 years old, no active use of tobacco, BMI < 30 Kg/m ^2^ , no active inflammatory pelvic disease in the year prior to IVF, and no presence of hydrosalpinx. The study group was comprised of infertile women with proven (laparoscopy and biopsy, at least 6 months prior to in vitro fertilization) endometriosis according to the American Society for Reproductive Medicine classification (ASRM) (1997). 17 The included patients presented only peritoneal endometriosis. The control group was comprised of infertile patients with tubal factor confirmed by laparoscopy. The exclusion criteria were: no evidence of autoimmune disease, patients negative for anti-thyroid antibodies, normal serum prolactin, thyroid-stimulating hormone, diagnosis of endometrioma or ovarian cyst, a poor response to ovulation induction, the presence of moderate/severe male factor, intracytoplasmic sperm injection during IVF treatment, and polycystic ovary syndrome. All patients were submitted to laparoscopy during their infertility investigation as previously mentioned.

### Ovarian Stimulation

An oestradiol-antagonist-recombinant follicle-stimulating hormone (FSH) protocol was used for ovulation induction in all patients. Briefly, 4 mg/day oestradiol was initiated on the 20th day of the preceding cycle. Gonadotropin was initiated at a standard dose of 225 UI of recombinant FSH (Puregon, Vetter Pharma-Fertigung GmbH & Co. KG, Ravensburg, Germany) applied subcutaneously (SC) on the second day of the current cycle. From the 6th day of recombinant FSH therapy onwards, the daily FSH doses were adjusted according to oestradiol levels and/or the number of growing follicles. Hormone serum levels of oestradiol, progesterone, and luteinizing hormone (LH) were evaluated, as was the follicular response, which was estimated by transvaginal ultrasound scans. The use of antagonist (Orgalutran, Vetter Pharma-Fertigung GmbH & Co. KG, Ravensburg, Germany) started on day 6 of the treatment cycle. When three or more follicles reached a size ≥ 17 mm, the final oocyte maturation was induced with a subcutaneous injection of human chorionic gonadotropin (hCG). Oocyte pick up under general anesthesia followed 36 hours later. Embryo transfer was performed on day 3 after insemination.

### Risk of Bias

We controlled the risk of bias using a strict eligibility and exclusion criteria as described before. Moreover, we performed a baseline group comparison and a multivariable analysis to detect any differences between both groups.

### Embryo Quality (Embryo Score)

**Fig. 1:**
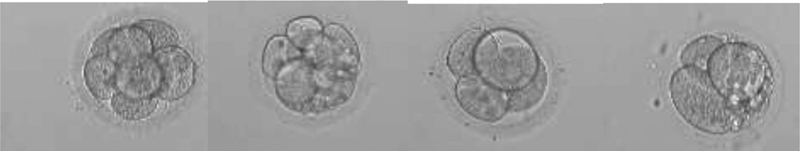
Embryos with 100, 80, 60, and 40 points according to the graduated embryo score.

The GES was performed based on three evaluations occurring at 16 to 18 hours, 25 to 27 hours and 64 to- 67 hours postinsemination by the same embryologist. The score was composed of the following criteria: nucleolar alignment along pronuclear axis, regular cleavage and degree of fragmentation at the first cell division, and cell number and morphology on day 3 after insemination. The mean score was calculated by the sum of the embryo scores divided by the number of obtained embryos, and the mean transferred embryo score was calculated by the sum of GES divided by the number of transferred embryos ( Fig. 1 ).

### Statistical Analysis

Absolute data were analyzed with the chi-square test or Fisher exact test. Continuous variables were compared using the Student *t* -test or analysis of variance (ANOVA). Multivariable analysis was done using linear or binary regression, controlling embryo score for endometriosis, body mass index (BMI), age, infertility length and number of retrieved oocytes. Data were analyzed using the SPSS Version 18.0 software package (SPSS Inc., Chicago, IL, USA). Significance was set at *p* < 0.05. Sample size was calculated from a pilot study conducted in 2009, and 70 patients were needed to have a 90% chance of detecting, at a 5% significance level, a decrease in the primary outcome measure from 92 in the control group to 85 in the experimental group. 18 In this pilot study, developed by Genro et al. (2009), 11 a GES value of 92 ± 12 was recorded in the control group without endometriosis, compared with 85 ± 9 in those infertile patients with endometriosis.

#### Ethics

This study was approved by the Ethical Committee of Hospital de Clínicas de Porto Alegre (IRB equivalent, #2002–0329). We utilized the Strengthening the Reporting of Observational studies in Epidemiology (STROBE) statement for observational studies. 19

## Results

**Fig. 2:**
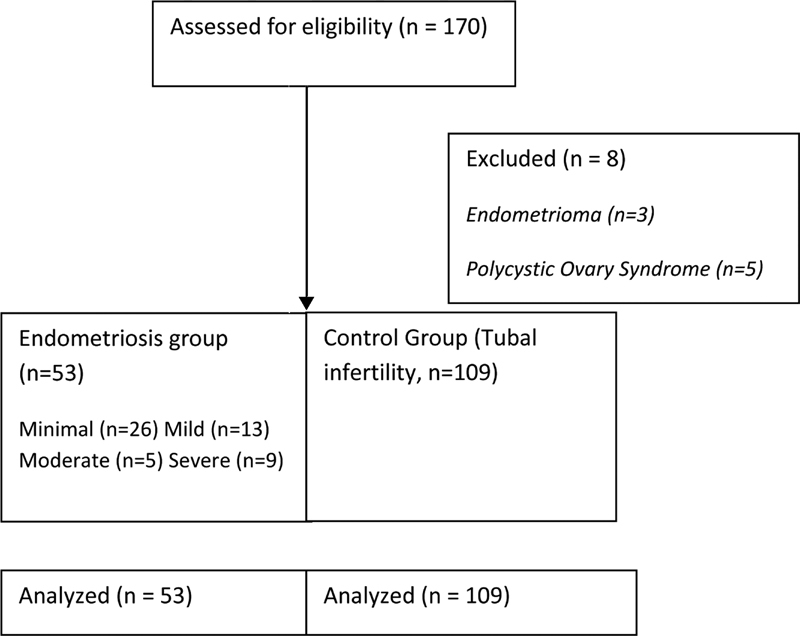
Study flowchart.

We assessed 170 eligible patients and excluded 8 (3 with endometrioma and 5 with polycystic ovary syndrome). Thus, we compared and analyzed 53 patients in the endometriosis group and 109 patients in the control group ( Figure 2 ). During the study period, 706 fertilized embryos were produced from 162 patients undergoing the IVF-ET procedure for infertility treatment. Of these embryos, 234 corresponded to patients with a diagnosis of endometriosis, and 472 corresponded to those from the control group. Of these embryos, 360 were transferred to the uterus on day 3 after insemination.

In the endometriosis group ( *n* = 53), 26 patients were classified as having grade I severity (minimal endometriosis), 13 as having grade II severity (mild endometriosis), 5 as having grade III severity (moderate endometriosis), and 9 as having grade IV severity (severe endometriosis). The clinical characteristics of both groups are shown in Table 1 . One thousand and nine oocytes were retrieved during the study period, of which 42% (424) were collected in the study group, and 58% (858) were collected in the control group.

**Table 1:** Subjects characteristics and laboratory/clinical outcomes of control and endometriosis groups (mean ± standard deviation) ( *n* = 162)

	Control group n = 109	Endometriosis group n = 53	*p* -value *
Age (years)	33.6 ± 5.61	33.0 ± 3.84	0.43
BMI (kg/m ^2^ )	23.6 ± 3.96	23.8 ± 2.93	0.80
Menarche (years)	12.5 ± 1.5	12.5 ± 1.2	0.90
Parity (n)	0.43 ± 0.82	0.17 ± 0.43	0.29
Abortion (n)	0.16 ± 0.46	0.15 ± 0.41	0.76
Time of infertility (months)	47.5 ± 45.3	48.5 ± 31.2	0.89
Mean GES AFC (n)	71.9 ± 23.5 11.6 ± 6.9	71 ± 19.8 11.5 ± 4.8	0.801 0.93
FSH (mUI/mL)	7.4 ± 4.6	6.4 ± 2.0	0.62
TSH (mUI/mL)	2.51 ± 1.77	2.28 ± 1.18	0.38
Prolactin (ng/mL)	17.9 ± 12.9	14.4 ± 7.3	0.50
Estradiol (pg/mL)	61.3 ± 43.2	46.5 ± 25.1	0.25
Total gonadotropin dose (UI)	2,634 ± 785.8	2,777 ± 711.8	0.27
Number of oocytes retrieved (n)	8.48 ± 4.2	8.38 ± 3.1	0.86

**Abbreviations** : AFC, antral follicle count; BMI, body mass index; FSH, follicle-stimulating hormone; Mean GES, mean graduated embryo score; TSH, thyroid-stimulating hormone.

* Student t-test.

Groups were compared according to their infertility characteristics (primary or secondary), with no significant difference being observed between groups ( *p* = 0.295). The abortion rate was similar in patients in the control group (12.8%, *n* = 14) and in those with endometriosis (13.2%, *n* = 7). There was no statistically significant difference for this endpoint ( *p* = 0.767).

The number of embryos obtained was similar in both groups (4.42/patient in study group and 4.33/patient in control group, *P* > 0.05). However, the number of transferred embryos in patients with endometriosis (2.38 ± 0.66) was significantly higher compared with the control group (2.15 ± 0.54; *p* = 0.001).

**Fig. 3:**
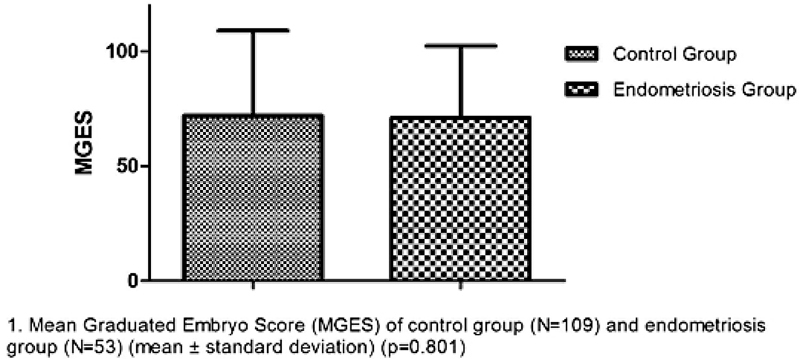
Mean graduated embryo score of control group and endometriosis group.

The mean GES of transferred embryos was 71 ± 19.8 in infertile patients with endometriosis and 71.9 ± 23.5 in patients from the control group (Graph 1), and there was no significant difference ( *p* = 0.801). When the different degrees of endometriosis were compared (ANOVA test), this study found the following scores: grade I (66 ± 18; *n* = 26/53); grade II (76.4 ± 24.7; *n* = 13/53); grade III (70 ± 9.2; *n* = 5/53); and grade IV (80 ± 19.6; *n* = 9/53), *P* > 0.05 ( Figure 3 ).

With regard to the GES of all embryos obtained, there was no difference when comparing the control and study groups. There was also no difference observed when comparing patients with endometriosis according to the degree of severity of the disease. The observed values according to different grades were as follows: grade I (62.3 ± 21); grade II (63.7 ± 19); grade III (63.8 ± 20); and grade IV (66.8 ± 16), with *p* = 0.945, ANOVA test.

To check for differences between the groups, we performed a secondary analysis considering that embryos with scores higher than 70 points have a higher implantation rate (11). Among the 109 control patients, 42 (38.5%) had mean GES scores greater than 70 compared with 20 patients from the 53 patients with endometriosis (37.7%). There was no significant difference between groups ( *p* = 0.888).

The fertilization rate was similar between both groups, corresponding to 61% of patients with endometriosis and 59% of the control group ( *p* = 0.511). Similarly, there was no difference between the study and control groups in terms of the cleavage rates, which were 89% and 90%, respectively ( *p* = 0.713). Among the patients with different degrees of endometriosis, the fertilization ( *p* = 0.976) and cleavage rates were also similar ( *p* = 0.432).

There was no statistically significant difference between patients with and without endometriosis with regard to their implantation rates, which corresponded to 21% and 22%, respectively ( *p* = 0.989). The pregnancy rates were (26% *vs* 28%) similar between the groups ( *p* = 0.853).

## Discussion

Several studies have suggested that endometriosis may impair fertility through different pathways, such as hormonal unbalance, oocyte dysfunction and endometrial receptivity. 6 7 20 We investigated if patients with this disease exhibit altered embryo quality when assessed based on the mean GES score system. We observed that the presence of endometriosis in infertile patients undergoing in vitro fertilization does not affect the mean GES. There were no previous reports in the literature comparing the use of GES between infertile patients with and without endometriosis (with tubal infertility). Moreover, we utilized the mean score because this is not affected by the embryo number. Therefore, the most important variable is the embryo quality, as measured by the mean score and not the number of embryos.

Altered embryo quality diagnosed by an abnormal embryo development and embryo blockage in patients with endometriosis has been described in the literature by several authors. 21 22 Moreover, these data have been supported by other studies on oocyte donation programmes. 13 However, several papers have reported that endometriosis does not affect embryo development or pregnancy rates. The most important and relevant aspect of endometriosis related infertility in patients submitted to in vitro fertilization is a drop in the ovarian reserve, causing a decreased number of oocytes after controlled ovarian stimulation for in vitro fertilization cycle. 3 8 9

Embryo selection for transfer is a critical step in in vitro fertilization treatment to optimize implantation rates and, finally, patient's pregnancy chance. Correctly evaluating the embryo quality before transfer is, therefore, of critical importance. 23 24 25 Thus, understanding if and how endometriosis affects the embryo quality may help clinicians in making everyday decisions.

In our population, although the number of embryos obtained was similar between the groups, we transferred a higher number of embryos in patients with endometriosis, as shown by others investigators. 16 Although we have found no difference in the implantation and pregnancy rates, the initial choice for this management was based on previous data, suggesting lower implantation and pregnancy rates in this group of patients. 5

The number of retrieved oocytes, fertilization rates, and total number of transferred embryos were similar in infertile patients with and without endometriosis. These findings were disputed by others with conflicting results. 3 9 26 The heterogeneity of patients, the difficulty in standardizing laboratory routines, and the presence of outcomes assessed in different ways between studies are limiting factors, and any results must, therefore, be critically evaluated.

Endometriosis has a wide spectrum of symptoms, and there is no consensus regarding how the different stages of the disease may affect women's fertility. Our population is composed predominantly of patients with minimal and mild disease, and we have not included patients with endometriomas. We have chosen to exclude patients with endometrioma to obtain an homogeneous and representative sample of patients with peritoneal endometriosis; however, it may have lowered our results and explain, at least partly, the lack of difference found in our sample. Moreover, patients with a poor response toward ovarian stimulation had their cycles cancelled before oocyte pickup and were excluded from our sample, as embryo quality analysis was not possible. This finding may mean that the most severe cases are not represented in our paper.

Furthermore, the embryo scores we have chosen to use to evaluate embryo competence involves considerable subjectivity. We have attempted to control for this factor by having a single observer perform all analysis in our study. The development of a scoring method for embryo evaluation on the third day after insemination or on blastocyst stage with higher reproducibility may be necessary to detect more subtle differences. The usefulness of comparing the mean embryo score instead of the total number of embryos per patient is to control for the effect of the individual number of embryos. 12

Our study has some flaws that we should discuss: a) we included only peritoneal endometriosis, which represents 70% of endometriosis phenotypes and, consequently, excluded the more severe forms of endometriosis; b) nowadays, embryo quality could be better evaluated using a time-lapse/artificial intelligence; however, this is a new and expensive technique for the majority of in vitro fertilization centers; c) our sample size was calculated using embryo score as the primary endpoint, but for comparisons of pregnancy or implantation rates our study was underpowered; d) the fact of including only peritoneal endometriosis could represent an early endometriosis presentation if compared with endometrioma. Therefore, we should analyze with caution our findings before extrapolating to all endometriosis phenotypes.

## Conclusion

In conclusion, the presence of peritoneal endometriosis in infertile patients submitted for IVF-ET did not affect embryo quality when measured by the mean GES. Further studies involving different scoring methods and mechanisms involved in the subfertility of patients with endometriosis are warranted.
